# The Interplay of COVID-19 and Hereditary Angioedema: Preventing an Acute Attack

**DOI:** 10.7759/cureus.29189

**Published:** 2022-09-15

**Authors:** Erin G Park, Zachary Silvano, Grant E Gregory, Mina Ghaly, James Case

**Affiliations:** 1 College of Medicine, Alabama College of Osteopathic Medicine, Dothan, USA; 2 Internal Medicine, Southeast Health Medical Center, Dothan, USA

**Keywords:** icatibant, covid-19 pneumonia, hae, hereditary angioedema, covid-19

## Abstract

Hereditary angioedema (HAE) is a rare inherited disease that is caused by the inactivation of the C1 esterase inhibitor. In this case report, we present a 51-year-old female previously diagnosed with HAE who tested positive for SARS-Cov-2 (COVID-19). The patient was treated symptomatically. Dexamethasone was used to treat COVID-19 pneumonia. Broad-spectrum antibiotics (vancomycin and meropenem) were utilized to prevent future infection. Although the patient did not experience an acute angioedema attack during her hospital stay, the patient expired due to the exacerbation of COVID-19 pneumonia.

## Introduction

Angioedema is characterized by swelling through dysregulation of histaminergic response or bradykinin, leading to an unchecked inflammatory cascade and vascular activation giving rise to vasodilation, increased permeability, and hypotension. SARS-Cov-2 (COVID-19) exhibits a similar mechanism by which des-arginine (9)-bradykinin (DABK), an analog of bradykinin acting on the same receptors, is left unchecked by depleted angiotensin-converting enzyme 2 (ACE2) [1]. Coincident activation of inflammatory pathways in an individual with both hereditary angioedema (HAE) and COVID-19 infection is described in this case report.

Studies show that infection of COVID-19 does not always lead to an exacerbation of HAE. In a study of 67 patients with HAE, only 10 patients (14.9%) were diagnosed with COVID-19. While the patients were infected with COVID-19, five out of 10 patients experienced HAE attacks [2]. The differentiating factor of patients with HAE is the level of C1 inhibitors present in the patient [3]. There are three different types that present in a clinical setting. Hereditary angioedema can be categorized as a consumption or inactivation mutation of the C1 esterase inhibitor (C1-INH). Type I HAE is the most common and is seen in around 85% of patients. These patients show a decrease in C1-INH production, while Type 2 shows normal or elevated CI-INH concentrations. Type 3 is extremely rare and will show normal levels and functions of C1-INH [4].

In our case report, a 51-year-old female previously diagnosed with HAE presented to the hospital with symptoms of respiratory distress. The patient subsequently tested positive for COVID-19 with a rapid antigen test. Short-term prophylaxis was successfully employed to prevent an acute angioedema flare. The case study will focus on the successful management of a patient with HAE that could have potentially been exacerbated with angioedema flares by COVID-19 pneumonia.

## Case presentation

A 51-year-old female with a past medical history of a C1 esterase inhibitor deficiency alongside hypertension, type-II diabetes mellitus (non-insulin-dependent), an ongoing seizure disorder, a recent history of deep vein thrombosis (DVT), and a known cerebrovascular accident (CVA) presented to the emergency with symptoms of dyspnea, a productive cough, nausea, vomiting, and fever. Her vital signs on arrival were as followed: Blood pressure (BP) 140/90 mmHg, a heart rate (HR) of 99, a temperature of 99.2 degrees Fahrenheit, and an oxygen saturation of 73% on room air. A rapid COVID antigen test revealed that she was positive for COVID-19. Suffering from acute hypoxic respiratory failure likely secondary to COVID-19 pneumonia, a central line was established and she was emergently intubated. The patient was admitted to the intensive care unit (ICU) for a full COVID-19 workup. 

After detailed history from the patient's family, it was revealed that the patient receives a monthly regimen of immunoglobulins combined with a C1 esterase inhibitor subcutaneous human injection (Haegarda®) 60 mg subcutaneously every three days for the prevention of angioedema related to her C1 esterase inhibitor deficiency. 

**Figure 1:**
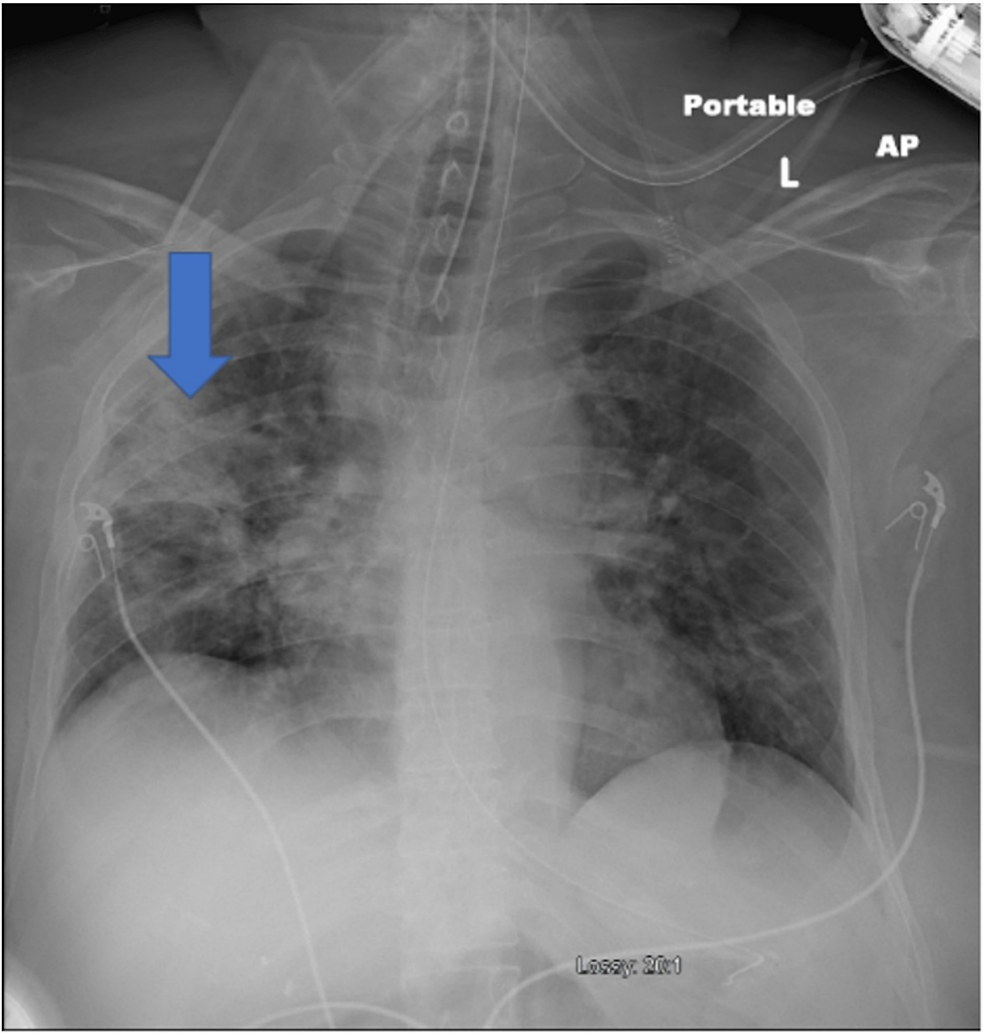
Endotracheal tube is at the level of the clavicular heads. Cardiac silhouette appears appropriate in size. No evidence of pneumothorax. Bilateral pulmonary opacities are present.

**Figure 2:**
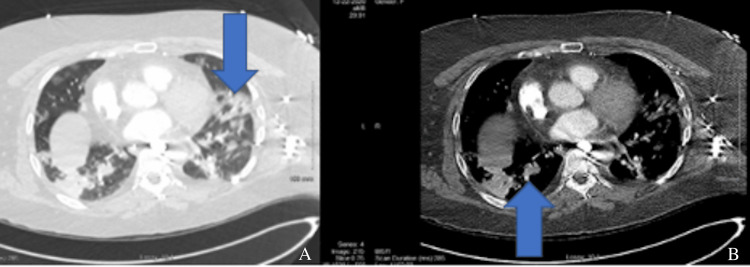
(A) and (B) are thoracic cavity CT scans that show bilateral extensive multilobar airspace disease, which is consistent with COVID-19 pneumonia.

**Figure 3:**
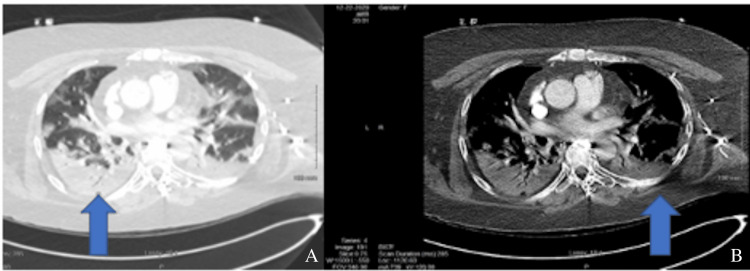
(A) and (B) are thoracic cavity CT scans that show bilateral extensive multilobar airspace disease, which is consistent with COVID-19 pneumonia.

A physical exam revealed course rhonchi in the anterior lobes bilaterally. Subsequent imaging revealed findings compatible with COVID-19 pneumonia, as evidenced by Figures 1, 2, 3. 

The plan for the patient was as followed: Treat COVID-19 pneumonia with dexamethasone at 6 mg every 24 hours through her central line and start broad-spectrum antibiotics, including vancomycin and meropenem. Vancomycin was administered through the central line with a loading dose of 30 mg/kg followed by a maintenance dose of 20 mg/kg every 12 hours. Meropenem was administered through the central line with a dose of 1 g every 12 hours. The patient also received DVT prophylaxis with apixaban 5 mg every 24 hours and seizure prophylaxis with levetiracetam 500 mg every 12 hours. 

Given the substantial risk of an angioedema attack from the acute stressors of intubation, an HAE short-term prophylaxis protocol would ensue [5]. The patient would continue her standard regimen of Haegarda®. Additionally, icatibant, a bradykinin 2 receptor antagonist, was kept at the bedside in case of an emergent angioedema attack. The dosage of the subcutaneous injection was 30 mg.

Throughout her ICU stay, the patient was continuously evaluated for laryngeal edema with cuff leak tests, which were negative. She was also evaluated for other signs of a pending angioedema crisis, including cutaneous and gastrointestinal manifestations such as skin rashes and changes in bowel habits. Those, too, were negative throughout her stay. 

Days later, the patient deteriorated and developed acute respiratory distress syndrome (ARDS) and worsening hypotension with unsuccessful spontaneous breathing trials due to COVID-19 pneumonia. The patient ultimately expired.

## Discussion

Hereditary Angioedema (HAE) is a rare autosomal dominant condition that leads to varying locations of swelling throughout the body with fluctuating severity. The cause of this condition is usually due to a genetic alteration in the C1-INH gene found on chromosome 11, also known as the Serpin Family Gene Member 1 (SERPING-1) gene, and 25% of patients may develop this condition sporadically [6]. As seen in Table 1, there are three types of this condition. Type 1 (HAE-1) has decreased production of C1-INH, while Type 2 has dysfunctional production of C1-INH, and Type 3 is considered a gain of function mutation in Factor XII. A deficiency in C1-INH is detrimental to the body's ability to regulate its fluid distribution. It inactivates the complement proteins C1, C2, and C4 of the complement cascade, which causes the classical pathway of the renin-angiotensin system to remain inactive [7]. An additional role of C1-INH is its ability to regulate coagulation factors such as XIIa and XIIf, which prevents the production of increased kallikrein [8]. With the deficiency of C1-INH, kallikrein is produced in excess, which leads to the production of kininogens and the release of bradykinin. This bradykinin binds to its type 2 receptor and causes an unregulated process that leads to edematous presentation in patients who suffer from HAE. Without the regulation of these proteins, it leads to excessive formation of anaphylatoxins that result in acute attacks localized to the skin and organs and eventually can lead to volume depletion in the body [8]. The defining characteristics of the edema seen in these patients are described as non-compressible, having no rash presentation, and vague margins around areas of swelling. Common locations that are affected by HAE flare-ups are the face, genitals, limbs, and visceral organ systems [8].

**Table 1:** Types of HAE The table is inspired from Duffey, Hannah & Firszt, and Rafael. (2015). Management of acute attacks of hereditary angioedema: Role of ecallantide. Journal of blood medicine. 6. 115-23. 10.2147/JBM.S66825.

Complement Factor Levels	Type I HAE	Type II HAE	Type III HAE
C4 concentration	Low	Low	Normal
CI-INH concentration	Low	Normal/High	Normal
CI-INH function	Low	Low	Normal

The 51-year-old female patient discussed within this report had a prior history of C1 esterase inhibitor deficiency, as well as a confirmed diagnosis of COVID-19 from a rapid antigen test. Developing HAE due to a COVID-19 infection or increased attacks of HAE during COVID-19 infection is rare. In a study examining the prevalence of HAE acute attacks with a COVID-19 infection, it was emphasized that in the study's cohort, all participants had a previous diagnosis of HAE Type I, II, or III, and their COVID-19 infections were symptomatic. Within the cohort, only 31% of the HAE patients had an attack during their COVID-19 infection. If an attack was reported, they were promptly treated with icabitant, a B-2 receptor antagonist, and reported the regions affected as the face, abdomen, and extremities [1].

The topic of whether an active COVID-19 infection can negatively impact those with a diagnosis of HAE is still being explored. The association is suggested in an article that states the virus enters host cells via the Angiotensin Converting Enzyme 2 (ACE2) receptor and depletes its capabilities of breaking down bradykinin. This inability for its usual function leads to possibly making the presentation of HAE more debilitating in those with an active COVID-19 infection [6]. The treatment that is utilized includes C1-INH replacement, lanadelumab, ecallantide, and icatibant [6].

**Figure 4:**
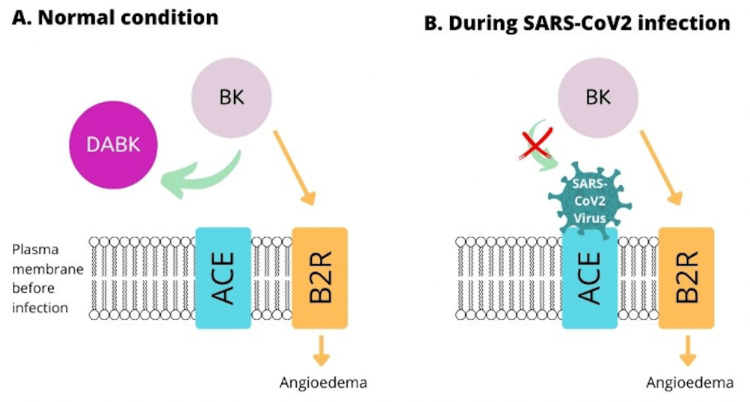
COVID-19's effect on ACE Figure is inspired from: Belbézie, A., Arnaud, M., Boccon-Gibod, I., Pelletier, F., McAvoy, C., Gobert, D., Fain, O., Du-Thanh, A., Launay, D., Lupo, J., & Bouillet, L. (2021, March 26). Covid‐19 as a trigger of acute attacks in ... - Wiley Online Library. COVID-19 as a trigger of acute attacks in people with hereditary angioedema. Retrieved August 4, 2022, from https://onlinelibrary.wiley.com/doi/10.1111/cea.13870

In this study, HAE patients showed an elevated number of T helper cells (Th17), and transforming growth factor (TGF-B). Patients with a COVID-19 infection showcased an elevated number of pro-inflammatory cytokines (IL-6, IL-17, and IL-21) as well as elevated T helper cells (Th17). While the use of icabitant as an antagonistic agent to the Kinin-Kallikrein system, ecallentide, a plasma kallikrein inhibitor, and lanadulemab, a monoclonal antibody against kallikrein, it has been suggested by researchers these treatments may assist in preventing the development of ARDS in COVID-19 patients [6]. There is a possibility that there is an repetitive activation cycle in patients with simultaneous infections of HAE and COVID-19 due to the viruses impact on ACE2, which increases levels of bradykinin (BK) and Des-Arg-9-Bradykinin (DABK) via the Kinin-Kallikrein system, but this needs to be explored further [6]. This interaction is seen in Figure 4.

An additional point was brought up by a study examining susceptibility to COVID-19 infection in those with diagnosed HAE. The questionnaire was completed by 1162 participants, of which 695 had confirmed HAE-C1INH (decreased C1-INH), and 175 participants had HAE-nl-C1INH (normal C1-INH). Results indicated insignificant findings between increased COVID-19 infection and HAE-C1INH. Statistical significance was seen with the HAE-nl-C1INH group and their likelihood of contracting COVID-19. In the patients with HAE-C1INH, who received prophylactic treatment with subcutaneous C1INH or Icatibant, there was a marked reduction in COVID-19 infection, while those with the HAE-C1INH and no prophylactic treatment had an increased risk of COVID-19 infection [9]. This complication of further COVID-19 exacerbation from a prior HAE infection is rare, so we are suggesting that the prophylactic treatment utilized for our patient played a part in preventing an HAE attack while she was recovering from her COVID-19 infection. In a study that reported the public's opinion on the impact that COVID-19 may have on HAE patients, it was expressed that providers and patients believe the medical treatment directed toward HAE will be negatively impacted by the burden COVID-19 has placed on the nation [10]. When analyzing this prevalence, there have been many concerns about an escalation in the severity of HAE attacks or even worsening the effects of the COVID-19 virus. In a study assessing the quality of life points for those with HAE within six months of diagnosis, It was found that half of their cohort experienced worse angioedematous attacks during their COVID-19 infection, while the other portion of the cohort reported insignificant findings that were in line with the control population [2].

Simultaneous infection with COVID-19 and HAE may lead to dire complications. HAE inflicts patients with sporadic edematous episodes alongside potentially life-threatening complications, and a reality where an increased susceptibility to COVID-19, or worse, increased severity of their symptoms, is a topic that needs to be further explored. Our case report is important in that we believe the prophylactic treatment, as well as continuing the patient’s already established treatment regimen, prevented her from having an HAE attack during her hospital admission for COVID pneumonia. Though the literature points towards a possible connection between this virus and disease, more research needs to be completed to cement a correlation between HAE and COVID-19.

## Conclusions

The pathogenesis of HAE overlaps with that of COVID-19, thereby elucidating potential mechanisms which may be affected to alter the natural course of the disease. Targeting inflammatory cascades and their constituents, such as bradykinin, may lead to a potent treatment for COVID-19 and prevent angioedema flares in the process.
